# Limited Bedding and Nesting Induces Maternal Behavior Resembling Both Hypervigilance and Abuse

**DOI:** 10.3389/fnbeh.2019.00167

**Published:** 2019-07-25

**Authors:** Meghan Gallo, Daniel G. Shleifer, Livea D. Godoy, Dayshalis Ofray, Aliyah Olaniyan, Talia Campbell, Kevin G. Bath

**Affiliations:** ^1^Department of Cognitive, Linguistic, and Psychological Sciences, Brown University, Providence, RI, United States; ^2^Physiology Department, Ribeirão Preto Medical School, University of São Paulo, São Paulo, Brazil; ^3^Department of Neuroscience, Brown University, Providence, RI, United States

**Keywords:** early life stress, early life adversity, limited bedding, abuse, kicking, maternal care, mouse model

## Abstract

Early life adversity (ELA) is associated with altered neural development and increased risk for the development of psychopathology across the lifespan. Rodent models of ELA are an important tool for investigating the possible mechanistic underpinnings of pathology development. We used a limited bedding and nesting model (LBN) to induce stress in the dam and alter dam-pup interactions during a sensitive period in early postnatal development. The primary characteristics previously identified in this model include fragmented and unpredictable maternal care and possibly neglect. However, previous studies have not considered the effects of this manipulation over the full circadian cycle and the evolution of changes of maternal behavior throughout the duration of the manipulation. In the current study, we leverage a novel continuous video monitoring setup to unobtrusively observe and subsequently analyze maternal behaviors. Through this more in-depth analysis, we discovered that LBN dams spent more time than control dams on their nest, returned to their nest more frequently than control dams, and showed intact maternal care. Importantly, a subset of LBN dams (~40%) engaged in abusive-like kicking, a behavioral pattern not previously identified in this paradigm. Exposure to ELA and abusive-like kicking were associated with differences in risk-taking behavior in adulthood. The LBN model of ELA may drive a more complex constellation of effects on maternal behavior driving a pattern of increased dam-pup interactions and increased abuse-like kicking behavior, with unique consequences for pup outcomes.

## Introduction

The type, amount, and predictability of parental care is crucial for supporting neural, cognitive and behavioral development (Harlow and Harlow, 1965; Harlow et al., 1965; Seay and Harlow, 1965; Caldji et al., 2000; Champagne et al., 2003; Rice et al., 2008; Murgatroyd et al., 2009; Bath et al., 2016). Attentive parental care and strong attachment promote a developmental profile that may buffer against the deleterious effects of stress and diminish risk for negative outcomes later in life (Suomi, 2006; Gee et al., 2014; Hennessy, 2014). Conversely, diminished quality of parental care, abuse and poor attachment, as well as increased parental stress, can alter hypothalamic-pituitary axis (HPA) development and reactivity in the offspring (Sanchez, 2006; Sanchez et al., 2010) and lead to increased lifetime risk for the expression of psychopathology, including depression, anxiety and post-traumatic stress disorder (PTSD; Dube et al., 2001). Deviations in the quality of parental care can come in a variety of forms, including multiple forms of abuse, neglect, anxious parenting and/or the loss of a caregiver. While it has been established that the accumulation of these and other forms of early life adversity (ELA) can increase the risk for a variety of forms of negative outcomes (Felitti et al., 1998; Brenhouse and Bath, 2019), how the different forms of altered parental care may confer unique risk for different forms of psychopathology are poorly understood.

Rodent models of ELA offer control over the type and timing of adversity while holding constant environmental and genetic factors. Here, we used a limited bedding and nesting (LBN) paradigm. In this paradigm, the dams access to adequate bedding and nesting materials was limited during the early postnatal development of her pups [postnatal day (P)4–11]. In previous reports, dams that reared pups in LBN conditions exhibited qualitatively similar features of maternal care in comparison to controls (licking and grooming, anogenital licking, etc.; Ivy et al., 2008; Rice et al., 2008; Bolton et al., 2017), but these interactions were unpredictable in timing and duration, and were termed *fragmented care* (Rice et al., 2008; Bolton et al., 2017). In turn, pups reared under LBN conditions exhibited biomarkers of altered HPA regulation early in life (Rice et al., 2008; Bath et al., 2016) indicative of ELA. Similar to outcomes associated with adverse childhood experiences in humans, the LBN rearing had long-term consequences on somatic and behavioral development, including delayed somatic growth (Ivy et al., 2008; Rice et al., 2008; Bath et al., 2016; Manzano Nieves et al., 2019), attentional impairments (Goodwill et al., 2018), increased depressive-like behavior (Goodwill et al., 2019), poorer spatial learning (Bath et al., 2017), delayed reproductive development (Manzano Nieves et al., 2019), accelerated maturation of select brain structures (Bath et al., 2016), and disruptions in threat-associated learning (Joëls et al., 2011; Bath et al., 2016; Manzano-Nieves et al., 2018). Collectively, these results imply that ELA in the form of LBN alters neural development with broader consequences for learning and affective responding across numerous domains.

A careful characterization of the impact of the LBN manipulation on maternal behavior is critical for placing findings from these studies in the appropriate context of the form of ELA experienced. To date, understanding the effects of LBN on maternal behavior has required either direct observation by an investigator of animals in their home cage in the vivarium (Ivy et al., 2008; Rice et al., 2008) or through the transport of animals to the lab to be recorded for short periods of time, followed by return to the housing room (Heun-Johnson and Levitt, 2016). Subsequently, behaviors have been hand scored based on a small number of hours during discrete periods of the manipulation. While these methods have provided important insights into the effect of LBN on maternal behaviors, they highlight that many factors may impact the investigator’s ability to observe the broader effect of the LBN conditions on behavior including: the consequences of investigator presence, disruption to the animals routine, behavioral scoring scheme, stress associated with animal transport, and the selection of hours to sample. These factors may also obscure the persistence or magnitude of group differences stemming from LBN manipulations or miss out on key changes in maternal behavior that can impact pup outcomes. For example, previous work from our lab has demonstrated that ELA in the form of LBN alters circadian cycling in mice in adulthood (Goodwill et al., 2018). Prior work in other species of rodents has shown that the preweaning period is critical for circadian entrainment of pups, with the dam playing a critical role in this process (Davis and Gorski, 1985). Thus, an understanding of the impact of LBN on the distribution of maternal behaviors over the circadian cycle has the potential to shed light on these later effects. Prior work has also shown that animals can both behaviorally and physiologically adapt to chronic adversity (McEwen, 2001). As the LBN manipulation is a persistent change in resources over a 7-day period, an understanding of the consequences of this manipulation on maternal behavior throughout the manipulation is critical. Here, we used a method to continuously and unobtrusively record mice in their home cage throughout the manipulation. We employed a combination of automated and hand scoring approaches across the circadian cycle and throughout the duration of the manipulation to more completely characterize the impact of LBN on maternal behavior. We hypothesized that the LBN would lead to circadian dependent differences in activity of the dams and that the nature of LBN dam-pup interactions would change throughout the manipulation.

We quantified the frequency and duration of maternal behaviors including eating, time spent on nest, and dam-pup interactions across the circadian cycle. Consistent with previous reports, we found that LBN manipulation increased the frequency of nest entries and exits. However, we found that LBN led to an increase in the duration of time spent on nest, a key marker of maternal care, an effect that was present throughout the circadian cycle. Furthermore, upon careful examination of the videos, we found that a subset of LBN dams engaged in a pattern of aggressive kicking and shoving behaviors directed toward the pups. Moreover, experiences of LBN and kicking predicted different adult behavioral phenotypes from those that resulted from LBN manipulations where kicking was not present. The current results indicate that less disruptive and more continuous means of tracking maternal behavior have the potential to provide more sensitive measures of changes in maternal behavioral. Further, we identify subgroups of animals in a given manipulation that engage in phenotypically different parenting styles in response to resource restriction, with potentially unique consequences on offspring development.

## Materials and Methods

### Mice and Housing

C57BL/6 mice were bred in house and maintained on a 12:12 light/dark cycle (lights on at 6:30, off at 18:30) with *ad libitum* access to food and water. Mice were housed in 31 (l) × 12 (w) × 14 (h) cm cages with bedding and a 4 × 4 cm cotton nestlet. Pups were weaned and segregated according to sex at postnatal day 21. All animal procedures and maintenance were in compliance with Brown University Institutional Animal Care and Use Committee and in accordance with the National Institutes of Health Guide for the Care and Use of Laboratory Animals.

### Limited Bedding and Nesting (LBN) Manipulation

Four days following the birth of a litter, dams and pups were transferred from their home cage into a 31 (l) × 31 (w) × 14 (h) cm cage with a wire mesh floor with a 4 × 2 cm cotton nestlet for 7 days [Postnatal day (P)4-P11]. Throughout this manipulation, mice continued to have *ad libitum* access to food and water. Following the limited bedding condition, dams and their pups were returned to normal bedding conditions.

### Maternal Home Cage Video Monitoring

**Figure 1:**
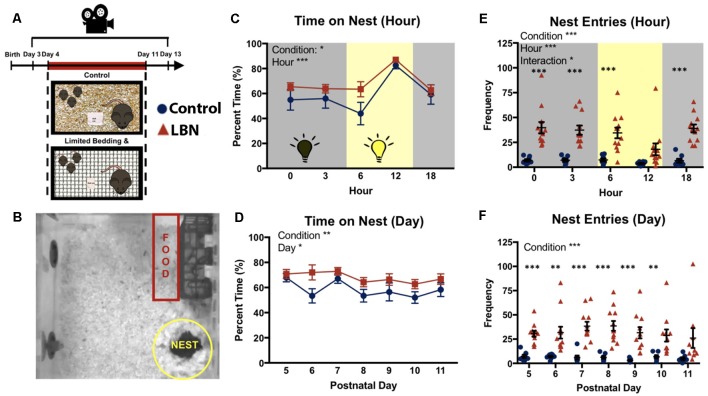
Effects of limited bedding and nesting (LBN) on nesting behaviors of dams. **(A)** Experimental timeline and setup. **(B)** Representative map of mouse home cage demarcating locations and size of nest and food zones. **(C)** Time spent on nest significantly differed by condition (*F*_(1,18.0)_ = 7.70, *p* = 0.01) and hour (*F*_(4,21.8)_ = 23.7, *p* = 1.16 × 10^−7^).** (D)** LBN dams spend significant more time on nest across days. Significant effects of condition were found at P6 (*t*_(17.5)_ = 2.24, *p* = 0.038) and trending at P10 (*t*_(13.0)_ = 1.81, *p* = 0.093). **(E)** Over the circadian cycle (dark = gray; light = yellow), a significant effect of condition (*F*_(1,17.8)_ = 29.67, *p* = 3.7 × 10^−5^), hour (*F*_(4,570.8)_ = 5.94, *p* = 1.1 × 10^−4^) and condition × hour interaction (*F*_(4,570.8)_ = 2.91, *p* = 0.02) were found for frequency of nest entries. **(F)** Across the 7 days of the manipulation, frequency of nest entries differed by condition (*F*_(1,17.40)_ = 29.73, *p* = 3.97 × 10^−5^), but did not significantly differ by day.

Mice in either LBN or control conditions were maintained in a specialized housing room in the vivarium, in typical housing cages [31 (l) × 31 (w) × 14 (h) cm] with a clear plexiglass cage topper with ventilation holes. Mice were transferred to the recording room in the vivarium when pups were P3, allowing a day or habituation to the recording room prior to LBN manipulations. Mice remained in this room for 10 consecutive days (P3–P13). Throughout this time, cages were undisturbed and continuously recorded with the aid of an overhead camera. Utilizing a combination of automated tracking and hand scoring, we observed dam-pup interactions in LBN and control cages across the circadian cycle (0:00, 3:00, 6:00, 12:00, and 18:00) throughout the duration of the manipulation (P4–P11). The remainder of videos were cataloged and can be made available upon request for further evaluation. Ethovision multiple body point tracking was used to quantify dam locomotor activity and location, specifically entries into and time spent in predefined zones including the nest, food, and water (Figure 1). Individual tracks were verified and edited for accuracy. On postnatal day 13, animals were removed from the recording setup and returned to standard housing.

### Hand Scored Assessment of Maternal Behaviors

To directly compare the selected circadian samples from the current study with previously reported methods for scoring of maternal behavior, we employed methods identical to those reported in Rice et al. (2008) and a slightly modified version from the procedure reported in Ivy et al. (2008). The modifications (inclusion of additional behaviors) was meant to provide a more detailed account maternal behaviors, including *kicking, carrying pups*, *exploring, digging* and *building nest*. Using continuous home cage video recordings, control (*n* = 6) and LBN (*n* = 12) dams were observed at times to reflect the changes in the circadian cycle (0:00 dark, 6:00 dark to light transition, 12:00 light, and 18:00 light to dark transition) at postnatal days 5 (Figure 3) and day 8 (Supplementary Figure S2) in order to be consistent with previous methods. Maternal observations occurred every other minute over the course of a 30-min period (described in Rice et al., 2008). The resulting 15-min epochs of maternal behaviors reflected the dams behavior: on (blue), off (red) and mixed (yellow; Rice et al., 2008; Figure 3A, Supplementary Figure S2A). Charts represent average behavior displayed within each epoch. Next, using a modified version of the Ivy et al. (2008) hand scoring protocol, the same videos were scored based on more detailed maternal behaviors: arched-back nursing (ABN; blue), self grooming (SG; green), licking and grooming pups (LG; yellow), carrying pups (turquoise), kicking pups (black), eating/drinking (pink), and building/digging (brown; Figure 3B and Supplementary Figure S2B). ABN reflected position of the dam, not the entire litter engaged in ABN. While it was possible to observe the number of LBN pups engaging in ABN, bedding and nesting materials obstructed observation of the number of control pups engaging in ABN.

**Figure 2:**
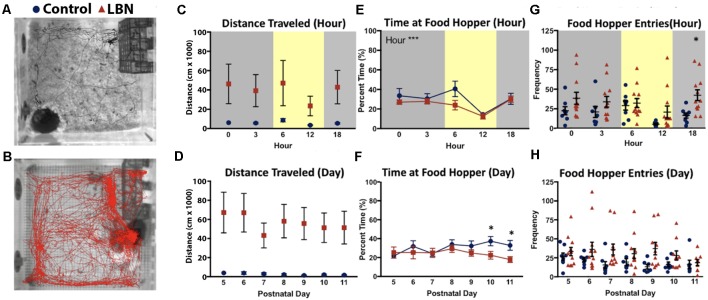
LBN rearing influences off nest behaviors of dams. Representative images of paths traveled by **(A)** control (black) and **(B)** LBN (red) dams for 1 h during the light cycle.** (C)** No main effect of condition, hour, or condition × hour interaction were found for distance traveled over the circadian cycle.** (D)** No main effect of condition, day, or condition × day interaction were found for total distance traveled across the 7-day manipulation. **(E)** A significant effect of hour (*F*_(4,27.1)_ = 14.58, *p* = 1.81 × 10^−6^) was found for percent time at food hopper, but no effect of condition or condition × hour interaction over the circadian cycle. **(F)** No main effect of condition, day, or day × hour interaction were found for time at the food hopper. However by days P10 and P11, controls spent more time at the food hopper than LBN dams. **(G)** For food hopper entries no main effect of condition, hour, or hour × condition interaction were found. However, at 18:00, LBN dams approached the food hopper more frequently than controls (18:00; *t*_(15.4)_ = 3.41, *p* = 0.004). **(H)** Across the 7 days of the manipulation, no main effect of condition, day, or day × condition effects reached significance for food hopper entries.

**Figure 3:**
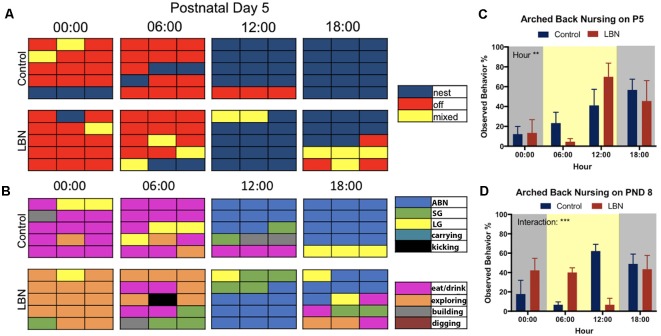
Comparison of current findings with previously used hand scoring approaches. **(A)** Representative graphs depicting the mean behavior expressed by each group during each epoch sampled. At postnatal day 5 (P5), control and LBN dams spent similar time on nest at 0:00, 6:00 and 12:00, with control dams having spent more time on nest that LBN mice at 18:00. **(B)** At hour 00:00 on postnatal day 5, control dams spent more time off nest and eating while LBN dams explored. At 06:00, LBN dams present more eating/drinking behavior than controls and a subset exhibited kicking behavior. At 12:00, all dams shifted behavior to arched-back nursing (ABN). At 18:00, control dams actively nursed while LBN dams engaged in mixed behavior. **(C)** Quantification of ABN demonstrated a significant effect of hour (*F*_(3,30)_ = 5.656, *p* = 0.003) but no condition or condition × hour interaction at P5. **(D)** At P8, a significant hour by condition interaction (*F*_(3,30)_ = 7.81, *p* = 0.0005) was found with LBN dams having engaged in more ABN during the transition from dark to light (6:00) and control dams engaged in more ABN than LBN dams at midday (12:00).

### Quantification of Nest Making and Proximity to Resources

Using home cage recordings, the total time to complete nest building was calculated from the time a dam was placed with a new nestlet until all pups were in one nest location with visibly fluffed cotton nesting (Supplementary Figure S1A). Nest proximity to resources was determined by the distance between the center of the nest and closest resource (food hopper or water bottle) using ImageJ 1.52a (Supplementary Figure S1B). All images were scaled based on the known size of the food hopper.

### Quantification of Maternal Kicking Behavior

**Figure 4:**
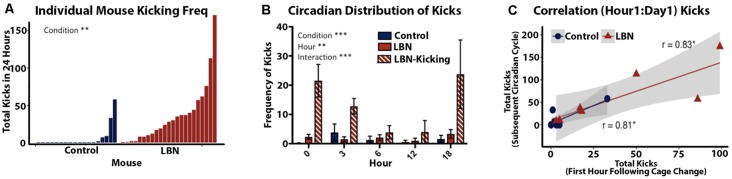
Quantification of LBN effects on frequency and predictability of abusive-like kicking behavior. **(A)** Quantification of kicking behavior for individual dams sampled from five selected time points over a 24 h period (P4–P5). Frequency of maternal kicking significantly differs by condition (*t*_(30.5)_ = 3.28, *p* = 0.0026). **(B)** Circadian distribution of kicking show a significant effect of condition (*F*_(2,159)_ = 22.94, *p* < 0.0001), hour (*F*_(4,159)_ = 3.624, *p* = 0.0074) and an hour × condition interaction (*F*_(2,159)_ = 22.94, *p* < 0.0001) with LBN dams engaging in higher kicking across the day. **(C)** A positive correlation was found between the first hour of kicking and frequency of kicking across the following day (*r* = 0.87, *p* = 2.1 × 10^−5^, *n* = 15). The first hour following cage change was highly predictive dam’s kicking phenotype for control (*r* = 0.81, *p* = 0.013, *n* = 8) and LBN dams (*r* = 0.83, *p* = 0.021, *n* = 7).

Kicking was defined as a dam’s forceful hindlimb or forepaw kick, visibly displacing the pup from the nest. Dam kicking behavior is overtly distinct from typical pup movement during nest building behaviors where the dam moves the pup with mouth and forepaws. Kicking was hand-scored during the first hour following a cage change (Figure 4C). Kicking behavior was also scored across the circadian span during the first 24-h of video recording, specifically postnatal day 4 at 18:00 and postnatal day 5 at 0:00, 3:00, 6:00 and 12:00 (Figures 4A,B). Due to high variability in kicking, we classified high kicking as dams that kicked more than 30 times over the selected time points during the first 24 h of video recording.

### Adult Outcomes

Between the ages of postnatal days 65–75, adult mice were tested on a behavioral battery consisting of the open field test, elevated zero maze, and light-dark box, with at least 1 day separating each test. Testing was performed in the same order, with the least invasive task (open field) being carried out first. Mice were categorized based on early life experience: (ELA; reared in LBN conditions), ELA with exposure to high kicking dam (ELA-K), and control.

### Open Field Test

Mice were placed in a 61 (l) × 51 (w) cm box under approximately 200 Lux of light under a digital camera for a total of 7 min. Noldus Ethovision XT 10 tracked mouse activity and location while in the apparatus. The open field was digitally divided into a peripheral 50% (outer zone), middle 25% (middle zone) and center 25% (center zone). Percent time in a defined zone was calculated based on the location of the center of mass of the animal.

### Elevated Zero

Mice were placed in a 33 (l) × 9 (w) cm circular maze consisting of two open and two enclosed sections (Shepherd et al., 1994) under approximately 250 Lux of light for a total of 7 min. To begin the trial, animals were place in the center of one of the open sections and were allowed to freely explore. Noldus Ethovision XT 11 tracked mouse activity using an overhead digital camera. Time spent in the open relative to closed arms, transitions, and total distance traveled were assessed. Arm time and entries were calculated based on transitions of the center mass of the mouse into a given zone.

### Light-Dark Box

The light-dark box test was carried out in a 58 (l) × 22 (w) cm box divided into two equal size light and dark sides connected by a 5 × 5 cm opening. Mice were allowed to freely move between sides. The light side was illuminated with approximately 2,150 lux of light and the dark side of the covered box was unilluminated (~10 lux). At the start of the 10-min trial, animals were placed in the dark side of the box. Noldus Ethovision XT 11 tracked mouse latency to enter the light side of the box, and then distance traveled, time spent in the light side, and entries into the light side.

### Analysis Strategy

For all dependent variables, we screened for statistical outliers prior to conducting further analyses and removed all observations that were more than 3 standard deviations above the raw, untransformed mean. Asterisks denote the following significance levels: *indicates a *p*-value less than 0.05, **indicates *p* less than 0.01, ***indicates *p* less than 0.001.

For analyses of Ethovision data over hour, we fit a linear mixed model with condition (LBN vs. control) and hour as predictors, treating condition as fixed effects, and hour as random effects (Figures 1, 3) and conducted an *F*-test on the fitted model. For analyses of Ethovision data over day, we used a linear mixed model by condition and day, treating condition as fixed effects, and day as a random effect (Figures 1, 3). Mixed-effects analyses were preferable to standard fixed-effects analyses because they appropriately assign variance across fixed and random factors. Additionally, mixed-effects analyses provided more flexibility in dealing with unbalanced designs while providing greater protection against Type I errors in comparison to fixed-effects analysis of variances (ANOVAs) with imputation^1^. To estimate mixed-effects regressions, we used the *lmer* function from the *lme4* package (version 1.1–17^2^), passing fitted models to the ANOVA function in base R (version 3.4.1^3^). We also conducted all *t*-tests using the *t*-test function in base R.

For analyses of nest making time and nest proximity to resources, we use unpaired *t*-tests to compare across condition on each measure (Supplementary Figures S2A,B). For analyses of kicking behavior, we fit a mixed linear model with condition and hour as predictors and conducted an *F*-test on the fitted model comparing the maternal behavior of kicking between groups across hour (Figure 4B).

Separate one way ANOVA was conducted to compare the effects of early life experience (by group: ELA, ELA-K, and control) on distance traveled and location on the open field test, distance traveled and location on the elevated zero maze, and latency to first entry to the light box and time spent in the light box on the light-dark box test.

## Results

Using an LBN paradigm to model ELA, we continuously recorded dams in their home cage from postnatal day P3 to P14 (Figure 1A). A subset of videos were selected across the circadian cycle and all days of the ELA manipulation (P4–P11). This provided the necessary temporal resolution to assess circadian effects on behavioral measures of mice without disrupting the animals daily routine or introducing stress associated with handling of cages or intrusion of the investigator. The remaining videos have been cataloged and can be made available for further analysis. Behavior was analyzed through a combination of commercial tracking software (Noldus Ethovision), to track the location of mice in the home cage (Figure 1B), and through hand scoring of select behaviors that were not amenable to quantification by automated systems.

### LBN Increases the Time That Dams Spend on Nest

To determine if LBN housing impacted the time that the dam spent in contact with her pups, percent time on nest was averaged across days for each sampled hour of the circadian cycle (0:00, 3:00; 6:00; 12:00; and 18:00 h). LBN dams spent a greater percentage of time on their nest compared to control dams (Figure 1C; *F*_(1,18.0)_ = 7.70, *p* = 0.01). Dams time on nest significantly differed across hours sampled (*F*_(4,21.8)_ = 23.7, *p* = 1.16 × 10^−7^), with dams spending the greatest amount of time on nest during the middle portion of the light:dark cycle. No hour × condition interaction was found (*F*_(4,21.8)_ = 0.56, *p* = 0.70). To determine if LBN conditions affected the percent time dams spent on the nest over the course of the LBN manipulation, data were averaged across hours for each day, and the effect of day was assessed. The main effect of rearing condition persisted (*F*_(1,17.9)_ = 8.76, *p* = 0.008), with LBN dams spending more time on nest than controls across the manipulation (Figure 1D). Upon visual inspection of the data, LBN dams appeared to spend significantly more time on the nest compared to controls at PND 6, however, the effect did not hold up following Bonferroni corrections for multiple tests (*t*_(17.513)_ = 2.25, *p* = 0.26). A significant effect of day was also observed (*F*_(6,572)_ = 2.62, *p* = 0.02), with dams spending less time with pups as the pups age. No day × condition interaction was observed (*F*_(7, 584.9)_ = 1.05, *p* = 0.40), indicating a similar shift in time on nest for both LBN and control dams across the testing period.

### LBN Leads to Increased Nest Entries/Exits

Previous studies have reported elevations in LBN dam nest “sorties” (exits from nest; Ivy et al., 2008; Rice et al., 2008; Heun-Johnson and Levitt, 2016). We first tested for effects of condition on nest entries/exits across the circadian cycle, collapsing data across days. Here, LBN dams had significantly higher rates of nest entries/exits compared to control dams (Figure 1E; *F*_(1,17.8)_ = 29.67, *p* = 3.7 × 10^−5^). *Post hoc* *t*-tests with Bonferroni corrections revealed significant effects of condition on nest entries at Hour 0 (*t*_(11.96)_ = 5.55, *p* = 6.35 × 10^−4^), Hour 3 (*t*_(13.141)_ = 6.2443, *p* = 1.4285 × 10^−4^), Hour 6 (*t*_(12.67)_ = 4.87, *p* = 1.66 × 10^−3^), and Hour 18 (*t*_(15.50)_ = 7.35, *p* = 1.0 × 10^−5^). An overall significant main effect of hour was also found (*F*_(4,570.8)_ = 5.94, *p* = 1.1 × 10^−4^), with both groups showing reduced nest entries/exits during the middle of the light:dark cycle, and a significant hour × condition interaction (*F*_(4,570.8)_ = 2.91, *p* = 0.02). Next, we collapsed across hour and tested for effects of day on this measure to determine if this effect was present across the entire manipulation. An overall effect of condition was observed (Figure 1F; *F*_(1,17.4)_ = 29.73, *p* = 3.97 × 10^−5^), with LBN dams continuing to show higher rates of nest entries/exits across the manipulation. No main effect of day (*F*_(6,31.6)_ = 0.59, *p* = 0.74) or day × condition interaction (*F*_(6,31.59)_ = 0.73, *p* = 0.63) was found, indicating that the frequency of dam nest entries/exists did not significantly change over the course of the manipulation.

### LBN Results in Increased Distance Traveled in the Home Cage

As LBN dams entered/exited the nest more frequently than control dams, we tested whether LBN dams moved around the home cage more than control dams. Using Noldus Ethovision to track the location of the dam, total distance traveled was computed for each hour and averaged across days (Figures 2A,B). When testing for circadian effects, the mean distance traveled by LBN dams was higher than controls. However, possibly due to high levels of variability in locomotion in LBN dams, this difference did not reach statistical significance (Figure 2C; *F*_(1,17.0)_ = 2.76, *p* = 0.12). Further, we did not observe a significant effect of hour (*F*_(4,29.9)_ = 1.02, *p* = 0.41), or hour × condition interaction (*F*_(4,29.9)_ = 0.81, *p* = 0.53) on distance traveled. To determine if differences in distance traveled existed over the course of the manipulation, distance traveled was collapsed across hour and effects of day were assessed. Over the course of the manipulation, distance traveled did not significantly vary by condition (*F*_(1,17.0)_ = 2.80, *p* = 0.11) or day (*F*_(6,41.7)_ = 0.55, *p* = 0.77) and no day × condition interaction was found (*F*_(6,41.7)_ = 0.31, *p* = 0.93; Figure 2D).

### LBN Alters Entries to but Not Time at Food Hopper

To determine if LBN conditions only impacted maternal behavior, or if it also impacted the number and duration of behaviors engaged in while off nest, we tested for effect of our manipulation on time and number of entries to the food hopper. To test for circadian effects on percent time at the food hopper, data were averaged across days for each hour. Over the circadian cycle, there was no main effect of condition, indicating that LBN dams spent a similar percentage of their time compared to controls at the food hopper (*F*_(1,16.9)_ = 2.51, *p* = 0.13). There was a significant main effect of hour (*F*_(4,27.1)_ = 14.58, *p* = 1.81 × 10^−6^), with animals spending a greater percentage of their time at the food hopper during the dark phase of the light:dark cycle, but no hour × condition interaction (*F*_(4,27.1)_ = 0.76, *p* = 0.56; Figure 2E). To determine if the percent time at the food hopper changed over the course of the manipulation, data were collapsed across hours for each day, and a complementary day × condition mixed-effects analysis was run. No effect of condition (*F*_(1,17.4)_ = 3.58, *p* = 0.08), day (*F*_(6,542.3)_ = 1.36, *p* = 0.23) or day × condition interaction (*F*_(6,542.3)_ = 1.38, *p* = 0.22; Figure 2F) were observed, indicating similar percentage of time at the food hopper for both groups over the course of the manipulation.

We also tested for effect of condition on food hopper entries to determine if LBN fragmented only dam:pup interactions or also fragmented visits to the food hopper (Figures 2G,H). For measures of hour, collapsing across days, no effect of condition (*F*_(1,18.0)_ = 0.62, *p* = 0.44), hour (*F*_(4,574.0)_ = 0.11, *p* = 0.98), or interaction were found (*F*_(4,574.0)_ = 1.35, *p* = 0.25; Figure 2G). However, upon closer analysis, exploratory follow-up *t*-tests with Bonferroni corrections revealed reliable differences in the later part of the day (18:00; *t*_(15.4)_ = 3.41, *p* = 0.02), when the frequency of control dam entries to the food hopper dropped to near zero and ELA dam entries remained relatively constant. To assess the effects of day, data were collapsed across hours. On average LBN dams made more entries to the food hopper than control dams (Figure 2H). However, due to high variability in LBN dam behavior, the effect of condition did not reach statistical significance (*F*_(1,17.6)_ = 2.94, *p* = 0.10). *Post hoc* *t*-tests with Bonferroni corrections did not reveal significant effects on any specific day. Further, there was no significant effect of day (*F*_(6,31.1)_ = 2.06, *p* = 0.09) or day × condition interaction (*F*_(6,31.1)_ = 1.02, *p* = 0.43).

### Nest Making and Nest Proximity to Resources

To determine if loss of resources impacted time to make a nest or location of the nest, these measures were also collected. LBN dams spent significantly less time making their nests in comparison to controls (*t*_(12)_ = 3.337, *p* = 0.005; Supplementary Figure S1C). There was no effect of condition on nest location (*t*_(16)_ = 1.25, *p* = 0.23) with LBN dams building nests at approximately the same distance from food or water sources as controls (Supplementary Figure S1D).

### Hand Scored Maternal Behaviors

Given that our manipulation utilized a more dense sampling approach and automated measures of dam location and nest entries, we wanted to compare the results of our manipulation to results obtained by other labs. To do this, we hand scored maternal behavior on days P5 and P8, using the methods described in (Rice et al., 2008; Figure 3A, Supplementary Figure S2A) and (Ivy et al., 2008; Figure 3B, Supplementary Figure S2B). We compared our findings (behaviors averaged within group at each epoch) to single animal representative graphs of previous studies.

At P5 using the protocol developed in Rice et al. (2008), LBN did not change how often mice were observed to be on nest (Figure 3A; blue) but led to a slight increase in observations of mixed behavior (Figure 3A; yellow). Using the scoring strategy reported in Ivy et al. (2008), differences between groups were more apparent (Figure 3B). LBN dams appeared to explore the cage more at times 00:00 and 06:00 (Figure 3B; orange) and dams exhibited kicking behavior at 6:00 (Figure 3B; black). Interestingly, in the current data, LBN dams appeared to show the most obvious differences during the transition to dark phase (18:00), but did not spend significantly less time on nest (*F*_(3,30)_ = 1.087, *p* = 0.3696; Supplementary Figure S2C). At this time point, control dams nursed on nest while LBN dams engaged in more periods of mixed behavior, intermixing nursing with eating (pink), licking and grooming (yellow), self-grooming (green), and exploring.

We also collected data using these same methods at P8 (Supplementary Figure S2). At the light transition (06:00), dams in the LBN conditions showed greater amounts of intermixed behaviors than controls, engaging in eating/drinking, mixed behaviors (yellow), nest periods (blue), self-grooming (green) and ABN (blue). Similar to what was observed at P5, control dams at 12:00 appeared to have spent more time in nest and engaged in greater amount of ABN than LBN dams. In contrast, LBN dams presented a fragmented pattern of behavior, spent more time engaged in off nest, mixed, and eating/drinking behaviors at P8. However, a mixed ANOVA on time spent on nest revealed no significant effects of condition (*F*_(1,10)_ = 0.5332, *p* = 0.4820; Supplementary Figure S2D).

To determine if LBN conditions altered the frequency at which dams were found to engage in ABN, frequency of this behavior was collected and analyzed. On P5, a significant effect of hour was found (*F*_(3,30)_ = 5.656, *p* = 0.003), but no main effect of condition (*F*_(1,10)_ = 0.00, *p* > 0.99) and no hour × condition interaction (*F*_(3,30)_ = 1.155, *p* = 0.34; Figure 3C). At P8, there was no main effect of condition (*F*_(1,10)_ = 0.02, *p* = 0.90) or hour (*F*_(3,30)_ = 1.785, *p* = 0.1713) for ABN; however, there was a significant hour × condition interaction (*F*_(3,30)_ = 7.81, *p* = 0.0005) with LBN dams having engaged in more ABN during the transition from dark to light (6:00) and control dams engaged in more ABN at midday (12:00; Figure 3D).

### LBN Leads to a Subset of Dams That Engage in Maternal Kicking

To this point, reports of LBN effects on maternal behavior have characterized the manipulation as a means of inducing fragmentation of care but had not identified any abusive behaviors. Using a more continuous and less intrusive means of recording maternal behavior, we found that a subset of LBN dams engaged in aggressive pup kicking behavior. To quantify this behavior, the frequency of maternal kicking was counted during 1-h intervals starting at 18:00 on P4 and then for 1-h epochs at 0:00, 3:00, 6:00 and 12:00 on P5. Total kicks during this period revealed a continuum with both LBN and control dams engaging in some kicking behavior. However, a significant effect of condition was observed (*t*_(30.5)_ = 3.28, *p* = 0.0026; Figure 4A) with kicking being far more common in LBN dams compared to controls. Based upon the observed distribution, we set a threshold to separate high kicking dams (LBN-K) from those where levels of kicking were low or absent. Dams that engaged in 30 or more kicks within the 5 h of observation were defined as high kickers. Using this criteria, 43% of LBN dams could be classified as LBN-K, while only 7% of control dams (a single dam) met this criteria. Using a mixed effects ANOVA, significant differences in pup kicking were found for condition (*F*_(2,159)_ = 22.94, *p* < 0.0001) and hour (*F*_(4,159)_ = 3.624, *p* = 0.0074) as well as an hour × condition interaction (*F*_(2,159)_ = 22.94, *p* < 0.0001; Figure 4B). To determine if there was a relationship between the number of kicks observed during the first hour of recording and the incidence of kicking during subsequent hours, we carried out correlation analysis. A positive correlation was found between the first hour of kicking and subsequent levels of kicking that were observed at later time points (*r* = 0.87, *p* = 2.1 × 10^−5^, *n* = 15). This indicated that initial kicking following a cage change predicted levels of kicking throughout the first full day of the manipulation. This positive correlation held up for both LBN dams (*r* = 0.83, *p* = 0.021, *n* = 7; Figure 4C) as well as controls (*r* = 0.81, *p =* 0.013, *n* = 8; Figure 4C).

### Adult Outcomes for Pups of Kicking and Non-kicking Dams

**Figure 5:**
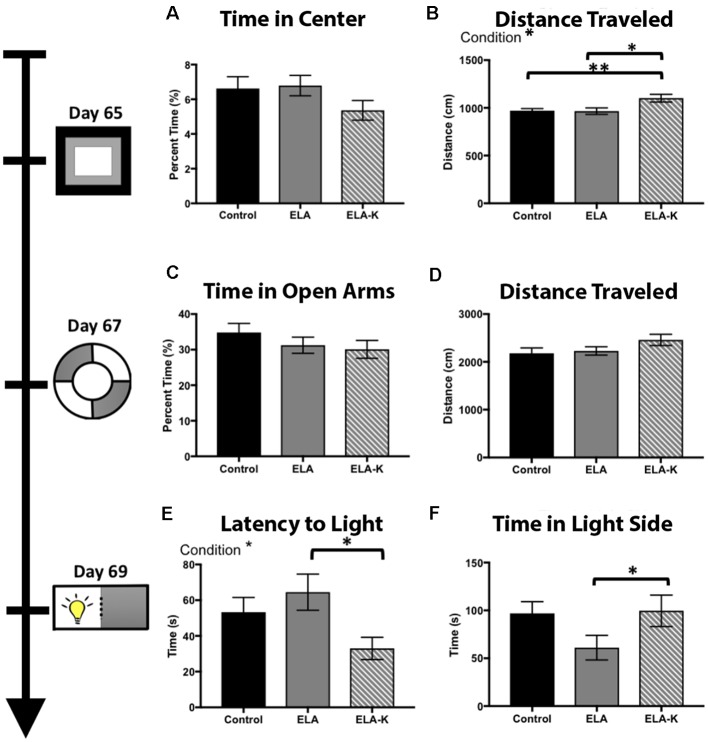
Effects of early life adversity (ELA) in the form of LBN rearing and LBN with kicking (LBN-K) on anxiety-like behaviors of adult offspring. **(A)** No significant effect of group was found for time in center of the arena (*F*_(2,118)_ = 0.77, *p* = 0.46) on the open field test. **(B)** LBN-K mice travel greater distance than other groups (*F*_(2,120)_ = 3.70, *p* = 0.03) on the open field test. **(C)** No significant effect of group was found for time spent in open arms of elevated zero maze (*F*_(2,1102)_ = 0.021, 73 *p* = 0.9918). **(D)** No significant effect of group was found for distance traveled on the elevated zero maze (*F*_(2,112)_ = 1.73, *p* = 0.18). **(E)** A significant effect of condition was found for latency to first enter the light side of the light:dark box (*F*_(2,66)_ = 3.176, *p* = 0.048). ELA-K adult mice entered significantly earlier than ELA animals (*t*_(34.5)_ = 2.644, *p* = 0.01). **(F)** No main effect of condition was found for time in light side (*F*_(2,71)_ = 2.43, *p* = 0.096), but ELA animals spend less time in the light compared to controls (*t*_(49.1)_ = 2.03, *p* = 0.048).

Given differences in the incidence and frequency of kicking behavior across LBN dams, we sought to test if differences in the incidence of kicking might impact adult anxiety-like behavior. LBN reared pups were divided into two groups, those reared under high kicking (ELA-K; >30 kicks) and low/no kicking LBN conditions (ELA; <30 kicks). To test whether early life experience including control, ELA and ELA-K differentially impacted anxiety-like behaviors in adulthood, mice completed a behavioral battery including the open field test (P65), the elevated-zero maze (P67), and the light-dark box (P69; Figure 5A).

### Open Field Test

We first compared adult behavior in the open field test using a one way ANOVA. Although we did not observe differences in time spent in the center of the maze vs. closer to the periphery (Figure 5A; *F*_(2,118)_ = 0.77, *p* = 0.46), we did observe clear differences in distance traveled between ELA-K, ELA and control reared pups (Figure 5B). A significant effect of condition was found for total distance traveled (*F*_(2,120)_ = 3.70, *p* = 0.03). *Post hoc* *t*-tests revealed that ELA-K adults traveled farther, overall, than both control adults (*t*_(29.9)_ = 2.87, *p* = 0.007), and ELA adults (*t*_(46.87)_ = 2.57, *p* = 0.01). Non-kicked ELA mice did not differ in distance traveled compared to controls (*t*_(84.76)_ = 0.12, *p* = 0.90). Thus, ELA-K adults differed in their general levels of activity from both control and ELA reared mice.

### Elevated Zero Test

In the elevated zero test, a one way ANOVA revealed no effect of condition for time spent in the open arms (*F*_(2,110)_ = 0.02 *p* = 0.99; Figure 5C). For total distance traveled, a one way ANOVA revealed no effect of condition on distance traveled (*F*_(2,112)_ = 1.73, *p* = 0.18; Figure 5D). ELA-K adults traveled farther than control reared adults. However, this effect did not reach statistical significance (*t*_(64.4)_ = 1.73, *p* = 0.09; Figure 5D). No differences were observed for distance traveled between ELA-K and ELA mice (*t*_(57.4)_ = 0.45, *p* = 0.12) or ELA and controls (*t*_(76.5)_ = 0.35, *p* = 0.72). While group differences were not as reliable as those observed in the open field test, the general pattern of greater movement by ELA-K adults was apparent in this task.

### Light-Dark Box

The light-dark box was used to assess anxiety-like behavior, as indexed by latency to enter the light side and time spent in the light zone (Figures 5E,F). One way ANOVA was used and group differences were found for both the time spent in light (*F*_(2,71)_ = 2.43, *p* = 0.096, Figure 5F) and latency to first entry (*F*_(2,66)_ = 3.18, *p* = 0.048, Figure 5E). Compared with ELA mice, ELA-K mice showed what appeared to be elevated risk-taking like behavior. First, ELA-K mice spent more time in the light side than non-kicked adults (*t*_(40.7)_ = 1.85, *p* = 0.07), and were indistinguishable from control animals (*t*_(40.8)_ = 0.13, *p* = 0.90). Here, non-kicked ELA mice spent less time in the light side of the box (*t*_(49.1)_ = 2.03, *p* = 0.048) compared with controls. Second, ELA-K entered the light side with a shorter latency than both ELA (*t*_(34.5)_ = 2.64, *p* = 0.01) and approached significance compared to controls (*t*_(44.4)_ = 1.96, *p* = 0.06). Thus, ELA-K adults appeared to show *reduced* anxiety-like behaviors, exhibiting more what appeared to be more risk-taking behaviors, as compared with both non-kicked ELA and control mice.

## Discussion

Here, we used a continuous video recording approach that allowed for undisrupted and unobtrusive observation of maternal behavior in the home cage throughout our manipulation. Using this approach, we built on the previous characterization of the effects of LBN on maternal behavior and expanded the window of observation to include the full circadian cycle and all days of the manipulation. We identified novel effects of LBN on dam:pup interactions (increased in LBN), found fragmentation in additional behaviors of LBN dams (food hopper entries), identified a subset of dams engage in abusive-like kicking behavior, and demonstrated differential outcomes on for pups reared in LBN conditions compared with LBN conditions with kicking. Moreover, we identified significant effects of circadian cycle and day on several of these behaviors, demonstrating that LBN effects on maternal behavior are not uniform across the day or manipulation. Factors of the circadian cycle and day should be taken into account when assessing biomarkers of risk/resilience in this model of resource restriction.

### LBN May be Driving a Hypervigilant Style of Parenting

Analyzing the pattern of dam behavior across the circadian cycle and across the manipulation allowed for a richer characterization of LBN effects on maternal behavior and activity. Utilizing continuous video recording of the home cage, we replicate the hallmark of the LBN manipulation on maternal behavior: increased nest entries/exits (Rice et al., 2008). Specifically, we observed an increased frequency of nest entries/exits across the circadian cycle and throughout the duration of the manipulation (Figures 1E,F). While previous studies have found that control and LBN dams spent similar time on nest (Ivy et al., 2008; Rice et al., 2008; Heun-Johnson and Levitt, 2016), we found that LBN dams spent more time on the nest compared to control dams across the circadian cycle (Figure 1C) and throughout the 7-day manipulation (Figure 1D). The ability to detect elevated time on nest in the current report may be the result of the continuous recording methodology and limited experimenter disruption associated with entering and exiting the room or movement of animals to recording setups. Further, the longer observation window allowed for sampling of more time points across the day and throughout the 7-day manipulation, possibly increasing the sensitivity of the current measures.

While we did find patterns consistent with fragmented care, increased nest entries/exits, the results were also consistent with evidence of *more contact overall* in LBN conditions. Rather than interpreting the observed results as an indication that dams left the nest more often, it is equally likely that LBN dams engaged in more attentive maternal behavior by more frequently returning to the nest and interrupting off nest behaviors. In an attempt to test this interpretation, the total duration and frequency of feeding bouts were assessed. While we were underpowered to detect significant main effects, there were significant effects of condition that emerged at select hours indicating a fragmentation in feeding behavior. Qualitatively observing the videos, it appeared that the elevation in nest entries/exits may in part be driven by dams returning to the nest more often to check on the pups, and disengaging prematurely from other behaviors, such as feeding, rather than failing to sustain contact with pups. Based upon these observations, it is plausible that this manipulation drives a form of stressed hypervigilant parenting associated with resources restriction.

To characterize possible differences in the quality of maternal care, the percent time engaged in ABN was assessed. Prior reports have found conflicting results (Ivy et al., 2008; Bolton et al., 2017) with LBN dams having either engaged in less ABN than control animals during the light cycle or the same rate as controls at postnatal days 5 and 8. Our results show that percent of ABN varies across the circadian cycle, and that observed effects may depend upon the time that this measure is collected. Here, the data indicates that LBN dams engage in similar overall levels of ABN to controls at P5 and a condition × hour interaction on P8, again indicating that the quality of maternal behavior is not diminished by LBN conditions.

### Identifying a Critical Need to Account for Circadian Effects on Maternal Behavior

Continuous, uninterrupted video recordings of maternal behavior can provide unique insights into the effects of LBN on maternal behavior over the circadian cycle. Here, significant interactions between condition and hour were found for a number of variables, including nest entries, indicating that the effects of LBN on these behaviors are not uniform across the circadian cycle. For this reason, restricting measurements to discrete time points, or averaging across time points, may mask important effects of this and other manipulations. For example, Rice et al. (2008) collapsed observations across 2 h in the light period (9:00 and 15:00) and 1 h during the dark period (20:00) while (Ivy et al., 2008) collapsed across 8:30 and 1:30 (light) and 18:30 (dark). Heun-Johnson and Levitt (2016) did not account for circadian influences and collected 4 h of video during the middle of the light phase of the animal’s cycle (12–4). Here, we collected data continuously, and sampled behavior during the light, dark, and transitions from light to dark and dark to light to allow for more detailed characterization of behavior across these periods. Many of the most robust differences were observed during the middle of the dark period (0:00, 3:00) and transition from dark to light (6:00). This approach identified periods of increased interest which can be further investigated using cataloged videos surrounding these periods. Further, by including more comprehensive measures of behavior across the light and dark cycle as well as light-dark transitions, may have also allowed for better characterization of effect rearing of the effects of condition across days. For example, we report a significant effect of day for time on nest (Figure 1D).

While circadian influences on maternal behavior may affect experimental observations, altered maternal behavior across the circadian span may impact pup development. Dams are critical in coordinating the circadian rhythms of their offspring in hamsters and mice (Davis and Gorski, 1985; Reppert and Schwartz, 1986). As LBN alters maternal behavior in a circadian dependent manner, activity shifts in the light/dark cycle may impact circadian entrainment of pups during sensitive developmental window, with possible lasting consequences. Indeed, recent work from our lab revealed that female LBN reared mice have disruptions in circadian patterns of resting and locomotor behavior, indicative of hypersomnia and depressive-like pathology (Goodwill et al., 2019). Future studies should explore how disruptions in circadian entrainment of dams in LBN may be impacting these outcomes, using emerging home cage monitoring techniques.

### LBN Increases the Incidence of Abusive-Like Maternal Kicking Behavior

While the LBN paradigm had been largely reported as a model of fragmented care and not maternal abuse, increased ultrasonic and audible pup vocalization have been reported when LBN dams were on nest (Heun-Johnson and Levitt, 2016). As pup ultrasonic vocalization may signal pain, these findings could be indicative of the presence of aggressive or abusive-like maternal interactions (Heun-Johnson and Levitt, 2016). Through analysis of video monitoring data, we observed a pattern of abuse-like behaviors that were almost exclusively expressed by LBN dams. Specifically, a subset of dams engaged in high levels of kicking of their pups immediately following a cage change. Importantly, this behavior persisted over the next 24 h at 18:00, 0:00, 3:00, 6:00 and 12:00 (Figures 4A,B). While a subset of LBN dams engaged in abusive-like behavior, kicking was rarely observed in controls. Interestingly, the likelihood and qualitative nature of kicking were distinct across housing conditions, but if present, they were immediate and robust. Specifically, kicks counted in the first hour following a cage change were highly predictive of the presence and frequency of kicks over the subsequent day (Figure 4C). Thus, a single hour of observation was indicative of which dams would kick their pups and to what extent. Given this finding, it will be important to track the effects of LBN on the expression of abusive-like behaviors, and simply sampling the first hour may provide a reliable index of levels of abuse over subsequent days. Studies using a different model of ELA in rats (Scarcity adversity), have found consistent elevations in abusive-like maternal care (Raineki et al., 2010; Perry et al., 2019). In that model, the degree and consistency of maternal abuse appear to be much greater than what is observed here. In the LBN model in mice, we observed variability in maternal care in response to resource restriction, with only a subset of dams engaging in kicking behavior. Thus, the LBN manipulation may allow investigators to tease apart two salient early life experiences (abusive-like experiences and resource restriction) following the exact same manipulation.

Natural variability in maternal care of rats during the first postpartum week, quantified by licking and grooming, has been shown to significantly impact offspring HPA, neural, cognitive and emotional development (Plotsky and Meaney, 1993; Caldji et al., 2000; Champagne et al., 2003). As variability in positive maternal care has profound implications for pup development, salient negatively-valenced maternal behaviors, including maternal abusive-like interactions can also profoundly impact pup development. The early postnatal period may be particularly sensitive to sensory inputs, particularly the type, timing, and salience of maternal interactions, with developmental consequences for brain and behavior. In other domains, exposure to harmful shock paired with neutral stimuli during early sensitive windows has lifetime consequences on the perception and valuation of the paired stimuli (Sullivan et al., 2000; Moriceau and Sullivan, 2006; Moriceau et al., 2009). Importantly, human exposure to ELA, increases lifetime risk for pathology including depression, PTSD, etc., with abuse specifically increasing the relative incidence of adolescent and adult risk for pathology (Kessler et al., 2010; McLaughlin et al., 2010; Roberts et al., 2015). Indeed, we demonstrated that pups exposed to ELA plus kicking exhibited differences in adult outcomes including engaging in what appears to be more impulsive risk-like behavior and increased locomotion (Figure 5), while ELA without abuse drove elevated risk assessment. Our results imply that maternal kicking in the LBN paradigm may constitute a model of abusive-like interactions with unique consequences on development. Future research is needed to understand the differential implications of resource restriction (ELA) and abusive-like interactions (ELA + Kicking) on neural and behavioral development and stress reactivity.

### Summary

The results reported here provide a comprehensive assessment of the effects of LBN on maternal behavior. Consistent with previous reports, LBN was found to drive elevations in dams entries/exits from the nest. However, dams exposure to LBN resulted in elevations in total time in contact with pups, no effects on levels of arched back nursing, and drove fragmentation of off-nest behaviors, effects that we interpret to represent a hypervigilant stressed parenting style. The ability to observe many of these phenotypic differences likely depends upon appropriate sampling the circadian cycle and days of the manipulation. Further, LBN housing was associated with increased risk for dams to exhibit abusive-like kicking behavior, which resulted in unique phenotypic outcomes for pups reared in LBN relative to LBN with kicking. Together these results provide a more nuanced picture of the effects of LBN housing on maternal behavior and highlights the potential importance of understanding the specific form that ELA takes when assessing phenotypic outcomes in these model systems.

## Data Availability

The datasets generated for this study are available on request to the corresponding author.

## Ethics Statement

All animal procedures and maintenance were reviewed and approved by Brown University Institutional Animal Care and Use Committee and in accordance with the National Institutes of Health Guide for the Care and Use of Laboratory Animals.

## Author Contributions

MG and KB conceived and designed the research. MG, DS, LG, DO, AO and TC collected the data. MG and LG analyzed the data. MG and KB generated the figures and prepared the manuscript.

## Conflict of Interest Statement

The authors declare that the research was conducted in the absence of any commercial or financial relationships that could be construed as a potential conflict of interest.

## Footnotes

^1^https://journals.sagepub.com/doi/abs/10.1177/009286150103500418

^2^https://cran.r-project.org/web/packages/arm/index.html

^3^https://www.r-project.org/
