# Supplementary material for: Limited Bedding and Nesting Induces Maternal Behavior Resembling Both Hypervigilance and Abuse
Source: Front Behav Neurosci. 2019 Jul 25;13:167. doi: 10.3389/fnbeh.2019.00167 (PMC6673755; doi:10.3389/fnbeh.2019.00167)
Supplement: Supplementary file 1 [file Data_Sheet_1.docx]

**Supplemental Information**

Differences in time engaged in nest construction and proximity of physical location of the nest to resources are two factors that may influence dams’ time on nest. For example, group differences for time on nest during the first day depend upon how much time that dam engages in nest building. With access to half of a cotton nestlet, LBN dams took approximately half of the time (about 50 minutes) to build their nest compared to controls (about 115 minutes) (**Supplemental Figure 1A)**. Given that nest were completed by 2 hours following the beginning of the manipulation, future differences in time on nest were not due to ongoing nest construction. To determine if increased time on nest or entries/exits from the nest might be due to differences in the relative location of the nest to resources (e.g. food or water), we measured the distance from the center of the nest to each of the resources. If control or LBN dams built their nest next to the food hopper, they would need to leave the nest less often for feeding. Conversely, if dams built their nest far from resources, they would spend more time off nest and increase distance traveled to eat or drink. Here, we found that the distance from nest to food and water were not statistically different between LBN and control dams, with LBN dams being marginally to resources than controls (**Supplemental Figure 1B**).

**Supplemental Figure 1**

**
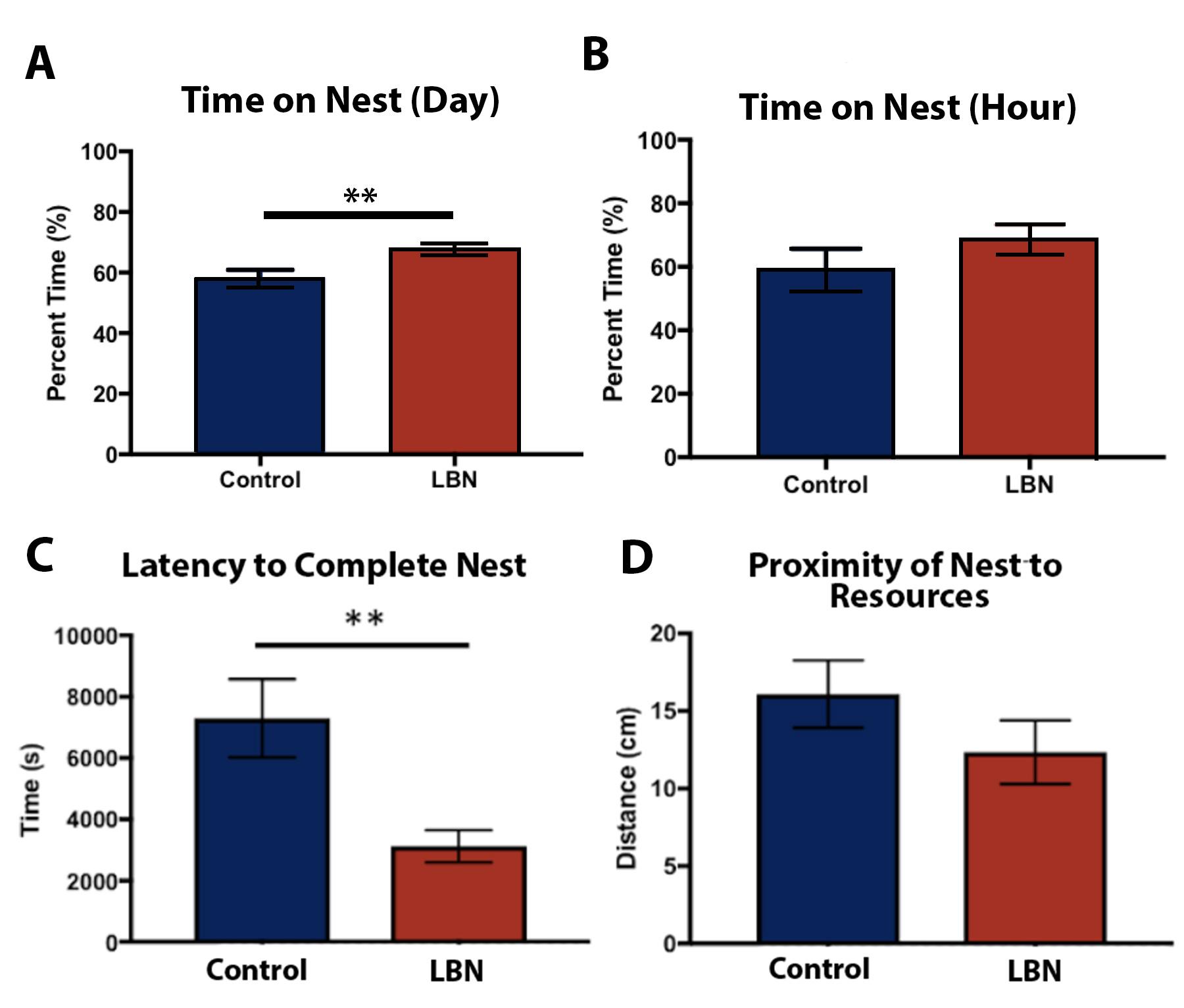
**

**Supplemental Figure 1:** **A)** LBN dams spent significantly more time on nest when averaged across days (*p* < 0.01) but, **B)** when averaged across hours the effect was trending toward, but did not reach significance (*p* = 0.06), suggesting that coarse aggregate measures may not have adequate sensitivity to detect circadian changes. *Effects of LBN housing conditions on nest building and location*: **C**) LBN dams made their nest significantly faster than controls (t_(12)_ = 3.337, *p* = 0.006). **D)** Dams do not exhibit a significant difference in nest proximity to resources of food or water between condition (t_(16)_ = 1.25, *p* = 0.229).

**Supplemental Figure 2**


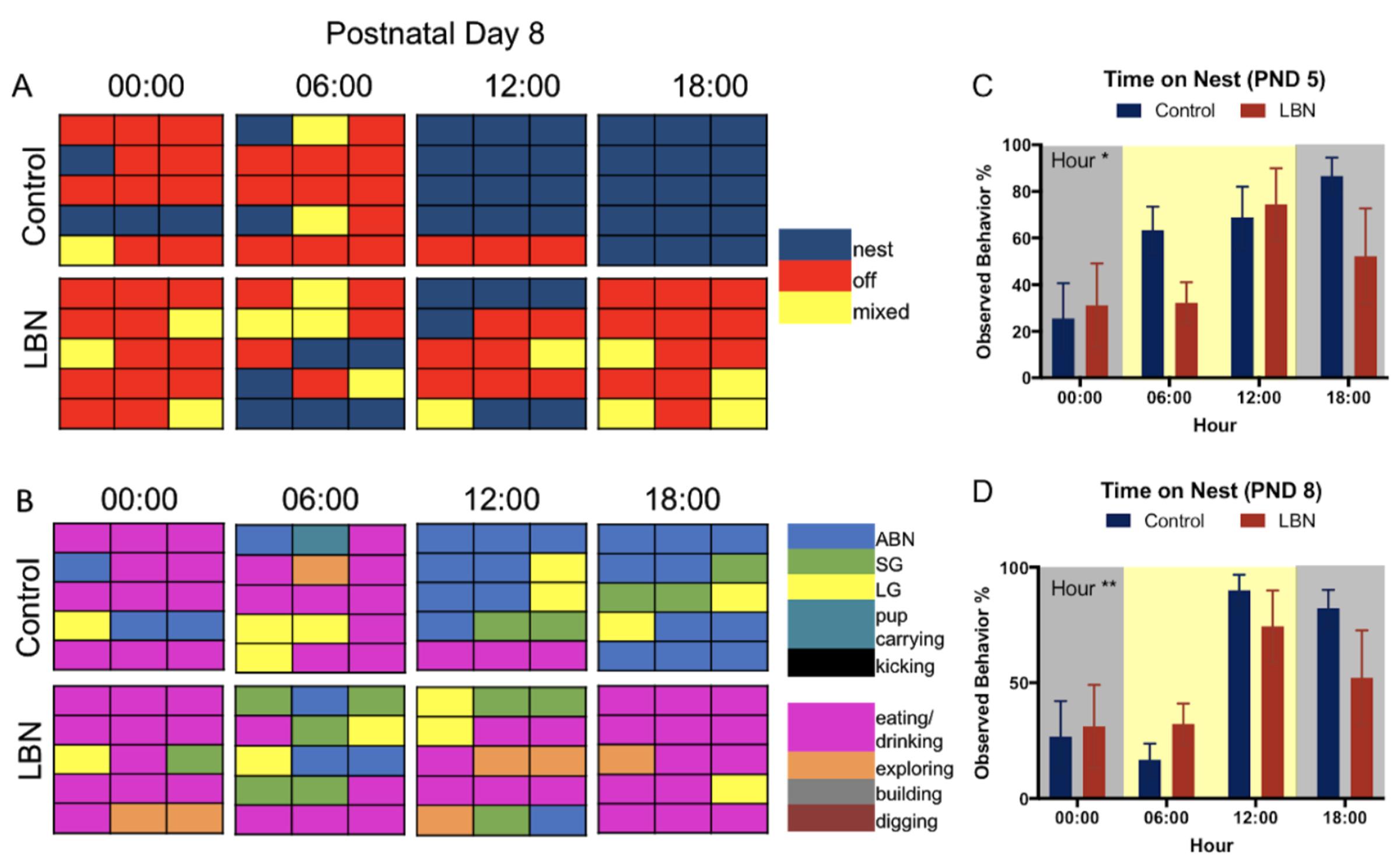


**Supplemental Figure 2:** Effects of LBN rearing on the distribution of hand scored behaviors at P8. **A)** Graphical representation of mean group behavior expressed at each epoch sampled. At hour 0:00, 12:00 and 18:00 on P8, control dams spent more time on nest compared to controls. At 6:00 LBN spent more time on nest than controls. **B)** On P8, both control and LBN dams similarly eat/drink at 0:00, but at 6:00 control dams spent more time eating/drinking than LBN dams. At 12:00 and 18:00, control dams spent more time on nest while LBN dams were eating/drinking. **C)** At P5, there is a significant effect of hour on time on nest (F_(3, 30)_ = 3.68, *p* = 0.023). **D)** At P8, there is a also significant effect of hour on time on nest (F_(3, 30)_ = 8.40, *p* = 0.0003).
